# Phenotypic clustering of atrial fibrillation patients using latent class analysis and its potential utility for ablation strategy: insights from the DIRECT-Extend and EARNEST-PVI studies

**DOI:** 10.1093/ehjopen/oeag135

**Published:** 2026-09-08

**Authors:** Shun Sasaki, Yuki Matsuoka, Akihiro Sunaga, Daisuke Sakamoto, Hideaki Hasegawa, Tomoharu Dohi, Hirota Kida, Tetsuhisa Kitamura, Katsuki Okada, Daisaku Nakatani, Hidetaka Kioka, Nobuaki Tanaka, Masaharu Masuda, Tetsuya Watanabe, Yasuo Okumura, Yoshiharu Higuchi, Koichi Inoue, Shungo Hikoso, Yohei Sotomi, Yasushi Sakata

**Affiliations:** Department of Cardiovascular Medicine, The University of Osaka Graduate School of Medicine, 2-2 Yamadaoka, Suita, Osaka 565-0871, Japan; Department of Cardiovascular Medicine, The University of Osaka Graduate School of Medicine, 2-2 Yamadaoka, Suita, Osaka 565-0871, Japan; Department of Cardiovascular Medicine, The University of Osaka Graduate School of Medicine, 2-2 Yamadaoka, Suita, Osaka 565-0871, Japan; Department of Cardiovascular Medicine, The University of Osaka Graduate School of Medicine, 2-2 Yamadaoka, Suita, Osaka 565-0871, Japan; Department of Cardiovascular Medicine, The University of Osaka Graduate School of Medicine, 2-2 Yamadaoka, Suita, Osaka 565-0871, Japan; Department of Cardiovascular Medicine, The University of Osaka Graduate School of Medicine, 2-2 Yamadaoka, Suita, Osaka 565-0871, Japan; Department of Cardiovascular Medicine, The University of Osaka Graduate School of Medicine, 2-2 Yamadaoka, Suita, Osaka 565-0871, Japan; Department of Social and Environmental Medicine, The University of Osaka Graduate School of Medicine, Suita, Osaka 565-0871, Japan; Department of Cardiovascular Medicine, The University of Osaka Graduate School of Medicine, 2-2 Yamadaoka, Suita, Osaka 565-0871, Japan; Department of Medical Informatics, The University of Osaka Graduate School of Medicine, Suita, Osaka 565-0871, Japan; Department of Cardiovascular Medicine, The University of Osaka Graduate School of Medicine, 2-2 Yamadaoka, Suita, Osaka 565-0871, Japan; Department of Cardiovascular Medicine, The University of Osaka Graduate School of Medicine, 2-2 Yamadaoka, Suita, Osaka 565-0871, Japan; Cardiovascular Center, Sakurabashi Watanabe Advanced Healthcare Hospital, 4-3-51 Nakanoshima, Kita-ku, Osaka 530-0005, Japan; Cardiovascular Center, Kansai Rosai Hospital, 3-1-69 Inabaso, Amagasaki, Hyogo 660-8511, Japan; Department of Cardiovascular Medicine, Yao Municipal Hospital, 1-3-1 Ryugecho, Yao, Osaka 581-0069, Japan; Division of Cardiology, Department of Medicine, Nihon University School of Medicine, 30-1 Oyaguchi Kami-cho, Itabashi-ku, Tokyo 173-8610, Japan; Cardiovascular Division, Osaka Keisatsu Hospital, 2-6-40 Karasugatsuji, Tennoji-ku, Osaka 543-0042, Japan; Cardiovascular Division, Hyogo Prefectural Nishinomiya Hospital, 13-9 Rokutanjicho, Nishinomiya, Hyogo 662-0918, Japan; Department of Cardiovascular Medicine, National Hospital Organization Osaka National Hospital, 2-1-14 Hoenzaka, Chuo-ku, Osaka 540-0006, Japan; Department of Cardiovascular Medicine, The University of Osaka Graduate School of Medicine, 2-2 Yamadaoka, Suita, Osaka 565-0871, Japan; Department of Cardiovascular Medicine, Nara Medical University, 840 Shijo-cho, Kashihara, Nara 634-8521, Japan; Department of Cardiovascular Medicine, The University of Osaka Graduate School of Medicine, 2-2 Yamadaoka, Suita, Osaka 565-0871, Japan; Department of Cardiovascular Medicine, The University of Osaka Graduate School of Medicine, 2-2 Yamadaoka, Suita, Osaka 565-0871, Japan

**Keywords:** AF, Latent class analysis, Catheter ablation, Oral anticoagulation

## Abstract

**Aims:**

AF is a heterogeneous syndrome. We aimed to assess whether latent class analysis (LCA) can identify phenotypes that stratify prognosis and predict differential responses to catheter ablation strategies.

**Methods and results:**

We first developed an LCA-based phenotyping model using the DIRECT-Extend registry (*N* = 7512), a pooled AF cohort of patients receiving anticoagulation therapy. Patients with echocardiographic data (*N* = 2741) were classified into latent clusters based on 16 clinical, laboratory, and echocardiographic variables. This phenotypic classification was then applied to the randomized Effect of Extensive Ablation on Recurrence in Patients with Persistent AF Treated with Pulmonary Vein Isolation (EARNEST-PVI) trial to evaluate whether the effectiveness of ablation strategies—pulmonary vein isolation (PVI) alone vs. PVI plus extensive ablation (PVI-plus)—varied across phenotypes. In the DIRECT-Extend registry, three phenotypes were identified: Phenotype 1 (*N* = 1565; ‘low comorbidity’), Phenotype 2 (*N* = 981; ‘cardiomyopathy & chronic kidney disease’), and Phenotype 3 (*N* = 195; ‘inflammatory-enriched AF’). Over a median of 600 days, the primary endpoint—a composite of all-cause death, acute myocardial infarction, any stroke, and major bleeding differed across phenotypes (log-rank *P* < 0.001). In EARNEST-PVI, patients were stratified into Phenotype 1 (*N* = 328), Phenotype 2 (*N* = 139), and Phenotype 3 (*N* = 30) according to the classification model. AF recurrence was significantly lower in the PVI-plus group compared to the PVI-alone group in Phenotype 1 (log-rank *P* = 0.013), whereas there was no significant difference in Phenotypes 2 and 3.

**Conclusion:**

LCA identified distinct AF phenotypes associated with prognoses. These phenotypes may also provide insights into heterogeneity in response to ablation strategies. This phenotyping approach may support risk stratification and individualized AF management.

## Graphical Abstract

**Graphical Abstract:**
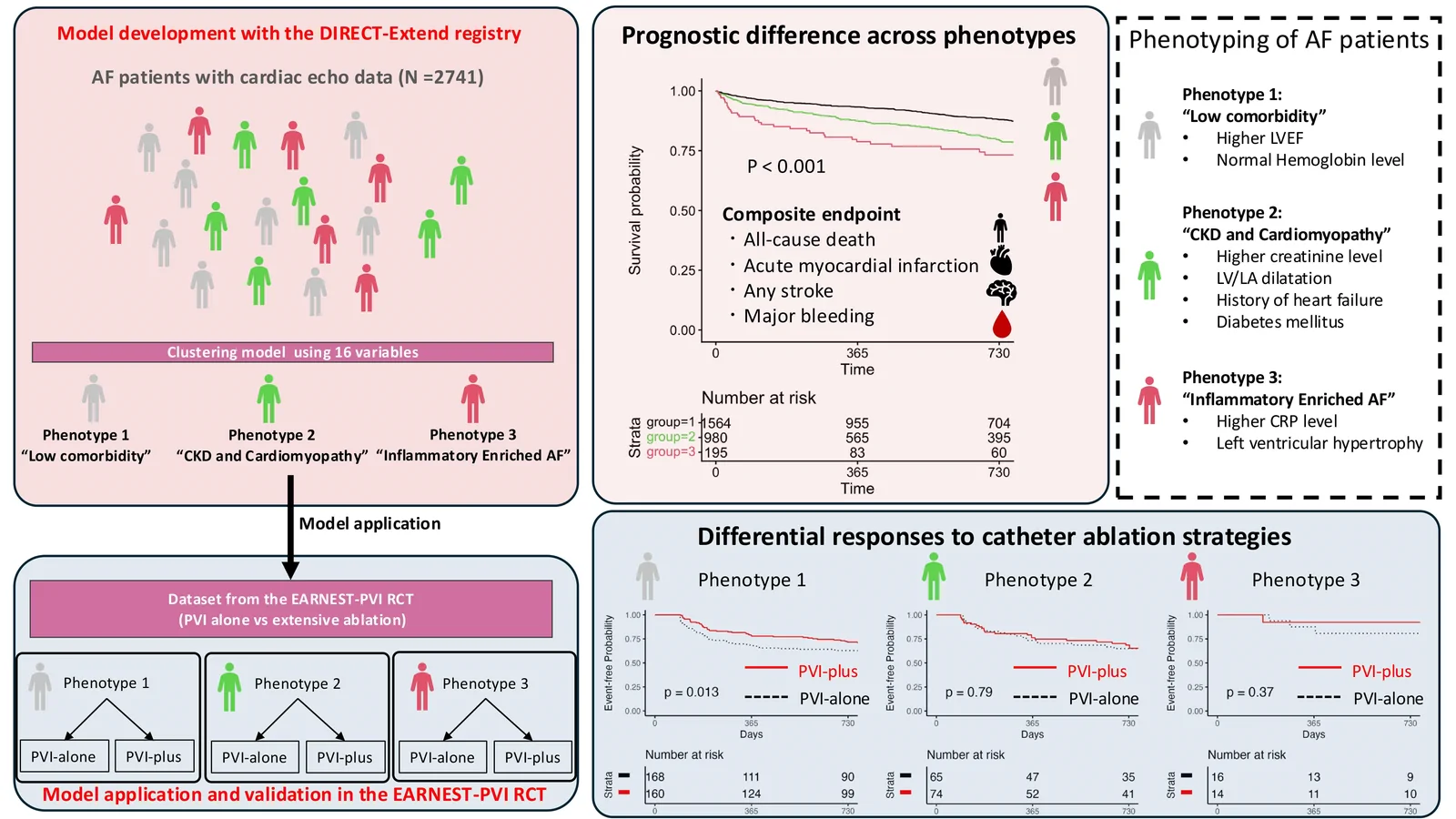

What’s new?

- Latent class analysis incorporating echocardiographic parameters allowed multidimensional risk stratification in a large AF cohort (DIRECT-Extend study).
- Application of this phenotyping to a randomized controlled trial (EARNEST-PVI trial) suggested variability in response to ablation strategies across phenotypes.
- Phenotyping may serve as a tool for both prognostic stratification and personalized treatment selection.

## Introduction

AF is a common and clinically significant arrhythmia associated with an increased risk of stroke, heart failure, and mortality.^1–3^ Conventional stratifications in AF patients have traditionally been based on a single clinical dimension, such as AF duration (paroxysmal, persistent, or permanent) or the presence of specific comorbidities represented by CHADS_2_ or CHA2DS2-VASc scores.^4,5^

However, AF is increasingly recognized as a heterogeneous syndrome resulting from the interplay of multiple systemic and cardiac factors, including structural remodelling such as atrial cardiomyopathy, metabolic and renal dysfunction, and lifestyle factors such as obesity and smoking.^1–3,6^ In clinical practice, physicians consider multiple clinical variables to capture the patient’s characteristics and determine appropriate treatment strategies. Conventional approaches limit the ability to stratify patients using various information and guide treatment decisions. Therefore, multidimensional data-driven approaches are needed to capture patient heterogeneity and inform personalized strategies.

Latent class analysis (LCA) enables multidimensional patient stratification by identifying latent subgroups from the observed clinical data. This method has gained traction in cardiovascular research, particularly in heart failure with preserved EF, where it has helped to organize heterogeneous patient characteristics, identify clinically meaningful subgroups, and assess risk stratification.^7,8^ Although clustering approaches have been applied to AF populations, most prior studies evaluated only prognostic differences.^6^ Few have examined whether these phenotypes are associated with differential treatment responses in the context of randomized prospective clinical trials.

One of the most important therapeutic strategies in AF management is rhythm control therapy with catheter ablation. Pulmonary vein isolation (PVI) is the cornerstone of ablation therapy. However, whether additional ablation beyond PVI—such as linear ablation or complex fractionated atrial electrogram (CFAE) ablation—provides incremental benefit remains controversial, particularly in patients with non-paroxysmal AF.^9^

The Effect of Extensive Ablation on Recurrence in Patients with Persistent AF Treated with Pulmonary Vein Isolation (EARNEST-PVI) trial was a multicentre, randomized study designed to address this unresolved clinical question by comparing the PVI-alone group with PVI plus empiric extensive ablation in patients with persistent AF.^10^ Although the trial did not demonstrate an overall superiority of either strategy, *post hoc* subgroup analyses suggested that the efficacy of extensive ablation beyond PVI varied substantially across patients, implying that treatment response is not uniform.

However, translating this heterogeneity into clinically actionable decision frameworks for selecting ablation strategies beyond PVI remains challenging. This gap likely reflects the limitations of traditional single-factor approaches in addressing the heterogeneity of AF. A multidimensional framework such as LCA may allow more precise stratification of AF patients for both prognostic evaluation and personalized treatment strategies.

In this study, we performed LCA in a large AF cohort from the DIRECT-Extend registry to identify clinically relevant phenotypes and applied these phenotypes in the EARNEST-PVI randomized trial to assess whether they were associated with differential responses to catheter ablation strategies.

## Methods

### Study population

This study utilized two large cohorts of patients with AF: the DIRECT-Extend registry and the EARNEST-PVI randomized trial. As these cohorts differ in design and objectives, the details of each study are described separately below. For the purpose of performing clustering analysis incorporating echocardiographic parameters, patients without echocardiographic data were excluded.

### DIRECT-extend registry

We established the DIRECT-Extend registry (UMIN000050585), a pooled database combining three large-scale observational studies of non-valvular AF (NVAF) patients treated with anticoagulation: the DIRECT prospective registry (*N* = 2539), the SAKURA-AF (Real World Survey of Atrial Fibrillation Patients Treated with Warfarin and Non-vitamin K Antagonist Oral Anticoagulants) prospective registry (*N* = 3237), and the Osaka University retrospective registry (*N* = 1736). Patients who visited or were admitted to the study institutions (Osaka University Hospital, Osaka Keisatsu Hospital, and 72 facilities participating in the SAKURA-AF registry) and were newly prescribed anticoagulants for NVAF were enrolled. The inclusion and exclusion criteria for the three registries have been described elsewhere.^11^ Briefly, in the DIRECT registry, all adult patients (aged ≥18 years) with NVAF at Osaka Keisatsu Hospital who initiated direct oral anticoagulant (DOAC) (dabigatran, rivaroxaban, apixaban, or edoxaban) from June 2011 to November 2021 were prospectively enrolled. In the SAKURA-AF Registry, all adult patients (aged ≥20 years) with a diagnosis of NVAF who were newly prescribed warfarin or DOACs for stroke prevention and had a follow-up duration of at least 1 year were prospectively enrolled between September 2013 and December 2015. Participating institutions included two cardiovascular centres (Nihon University Itabashi Hospital and Nihon University Hospital), 13 affiliated or community hospitals, and 48 private clinics, all located mainly in the Tokyo metropolitan area.^12^

### EARNEST-PVI trial

This study analysed extended data from the EARNEST-PVI trial (ClinicalTrials.gov, NCT03514693). The original EARNEST-PVI trial was a prospective, multicentre, randomized, open-label trial comparing 1-year rhythm outcomes between two ablation strategies, PVI alone (PVI only) and PVI plus (linear or CFAE ablation in addition to PVI), in patients with persistent AF. The study was performed by the Osaka Cardiovascular Conference Arrhythmia Investigators. The study design and primary results have been reported previously.^10^ Patients were enrolled across eight high-volume centres specializing in catheter ablation for AF. Eligible patients were those undergoing initial catheter ablation for sustained AF episodes lasting ≥7 days and <5 years. Exclusion criteria were as follows: left atrial diameter ≥50 mm, valvular AF, prior cardiac surgery, maintenance haemodialysis, LV EF <30%, and New York Heart Association class ≥3 heart failure. All patients provided written informed consent to participate, and the study was approved by the ethics committee of each hospital. After informed consent was provided, patients were randomized to either the PVI-alone group or the PVI-plus group.

### Clinical variables

The most recent available data within 6 months prior to the enrolment date were used in this study. If multiple measurements were available, the data closest to enrolment was selected. Clinical variables included clinical characteristics, comorbidities, laboratory data, and echocardiographic parameters. Clinical variables were collected as part of routine clinical practice at each participating centre.

### Clinical outcomes

We applied LCA to the dataset from the DIRECT-Extend registry to identify clinically relevant phenotypes and examine their prognostic implications. The primary endpoint was a composite of all-cause death, acute myocardial infarction, any stroke, and major bleeding according to the International Society on Thrombosis and Haemostasis criteria.^13^ The secondary endpoints were the individual components of the primary endpoint, clinically relevant non-major bleeding defined according to the International Society on Thrombosis and Haemostasis criteria,^14^ any bleeding which is a composite of major bleeding and clinically relevant non-major bleeding, ischaemic stroke, haemorrhagic stroke, systemic embolism, and heart failure admission.

We applied the phenotyping model derived from the DIRECT-Extend registry to the EARNEST-PVI randomized trial to assess whether the effect of extensive ablation strategy on AF recurrence varied across phenotypes. The primary endpoint of the EARNEST-PVI trial was recurrence of AF, which was defined as documentation—by 12-lead electrocardiogram or other appropriate diagnostic tools—of any atrial arrhythmia, including AF, atrial flutter, or atrial tachycardia, lasting longer than 30 s. A blanking period of 3 months was implemented. An independent events evaluation committee, blinded to group allocation, adjudicated all suspected recurrences.

### Statistical analysis

The machine-learning-based unsupervised cluster analysis and other statistical analyses were all performed using R software (version 4.4.2; R Foundation for Statistical Computing, Vienna, Austria). A *P* value < 0.05 was considered statistically significant. We applied the machine-learning-based unsupervised cluster analysis, LCA, to the DIRECT-Extend dataset. A total of 173 variables at registration were considered as primary candidates for the LCA. Covariates covered a wide range of domains, including patient demographics, clinical variables, laboratory data, and echocardiographic parameters. Among these, 111 variables were excluded: 26 variables contained prognostic information not available at baseline, 28 represented medication data, and 57 were not collected in the EARNEST-PVI trial. For the remaining 62 variables, those with ≥20% missing data (*N* = 16) were excluded. Random forest imputation using the ‘missForest’ package was performed for the remaining variables prior to variable selection and clustering.^15,16^ Variables with a correlation coefficient >0.6 were filtered by retaining the variable that was most clinically relevant and informative. Finally, a total of 20 continuous and categorical variables were entered into the LCA, resulting in 16 variables being retained in the final phenotyping model (see Supplementary material online, *Table S1*). We used the ‘VarSelLCM’ package (version 2.1.3.2) in R (version 4.4.2) to try between 2 and 10 clusters to identify the optimal number of clusters and most relevant discriminatory variables. In general, the optimal number of phenotypes within the cohort using the model-based clustering can be determined with optimization of the Bayesian information criterion (BIC). The BIC introduces a penalty term for the number of parameters in the model to avoid overfitting of the model created. The optimal number of clusters is the model with the lowest BIC value. We computed the discriminative power of each variable as the logarithm of the ratio between the probability that the variable is relevant for clustering and the probability that it is irrelevant for clustering. Because the clustering algorithm models continuous variables without assuming normal distributions and is robust to skewed data, continuous variables, including C-reactive protein (CRP), were analysed on their original scale without log transformation. This study followed the Transparent Reporting of a multivariable prediction model for Individual Prognosis Or Diagnosis + Artificial Intelligence (TRIPOD + AI) reporting guidelines and the European Heart Rhythm Association Artificial Intelligence (EHRA AI) checklist. The TRIPOD + AI checklist is provided in Supplementary material online, *Table S2*, and the EHRA AI checklist in Supplementary material online, *Table S3*. A detailed description of selected methodological components in accordance with these guidelines is provided in the Supplementary Methods.

## Results

Among 7512 patients initially enrolled in the DIRECT-Extend registry, 4771 patients without echocardiographic data (including all patients from the SAKURA-AF registry and those with missing echocardiographic data in the DIRECT and Osaka University registries) were excluded, leaving 2741 patients for the final analysis (*Figure 1*). Patient characteristics and clinical outcomes in the included and excluded groups based on echocardiographic data availability are presented in Supplementary material online, *Table S4*. As described in the Methods section, after stepwise exclusion of features with significant missingness or high correlation, a total of 20 features were used for the unsupervised machine learning-based cluster analysis (LCA) (*Figure 1*, Supplementary material online, *Table S1*). In this analysis, we observed a trend where the BIC decreased with an increasing number of clusters. However, with more groups, the clustering becomes less practical for everyday clinical use, and interpreting the characteristics of each group becomes challenging. Based on the Elbow method, which identifies the point at which further increases in the number of clusters provide slight improvement in model fit, and considering both clinical applicability and interpretability, we determined that clustering into three groups was most appropriate (see Supplementary material online, *Figure S1*). The LCA selected 16 variables for the optimal model. These variables were summarized in *Figure 2* with their discriminative power for the top 10. The most discriminatory variable was CRP, followed by interventricular septal thickness in diastole, LV EF, creatinine, and LV end-diastolic diameter. The probability of misclassification is illustrated in Supplementary material online, *Figure S2*, showing that the misclassification rate seems negligible. To assess the potential impact of imputation on the clustering results, we performed a sensitivity analysis by repeating the clustering using the original dataset without prior imputation, allowing the VarSelLCM algorithm to internally handle missing values. Although minor differences in cluster composition were observed, the overall phenotypic patterns remained largely consistent with the primary analysis (see Supplementary material online, *Table S5*).

**Figure 1:**
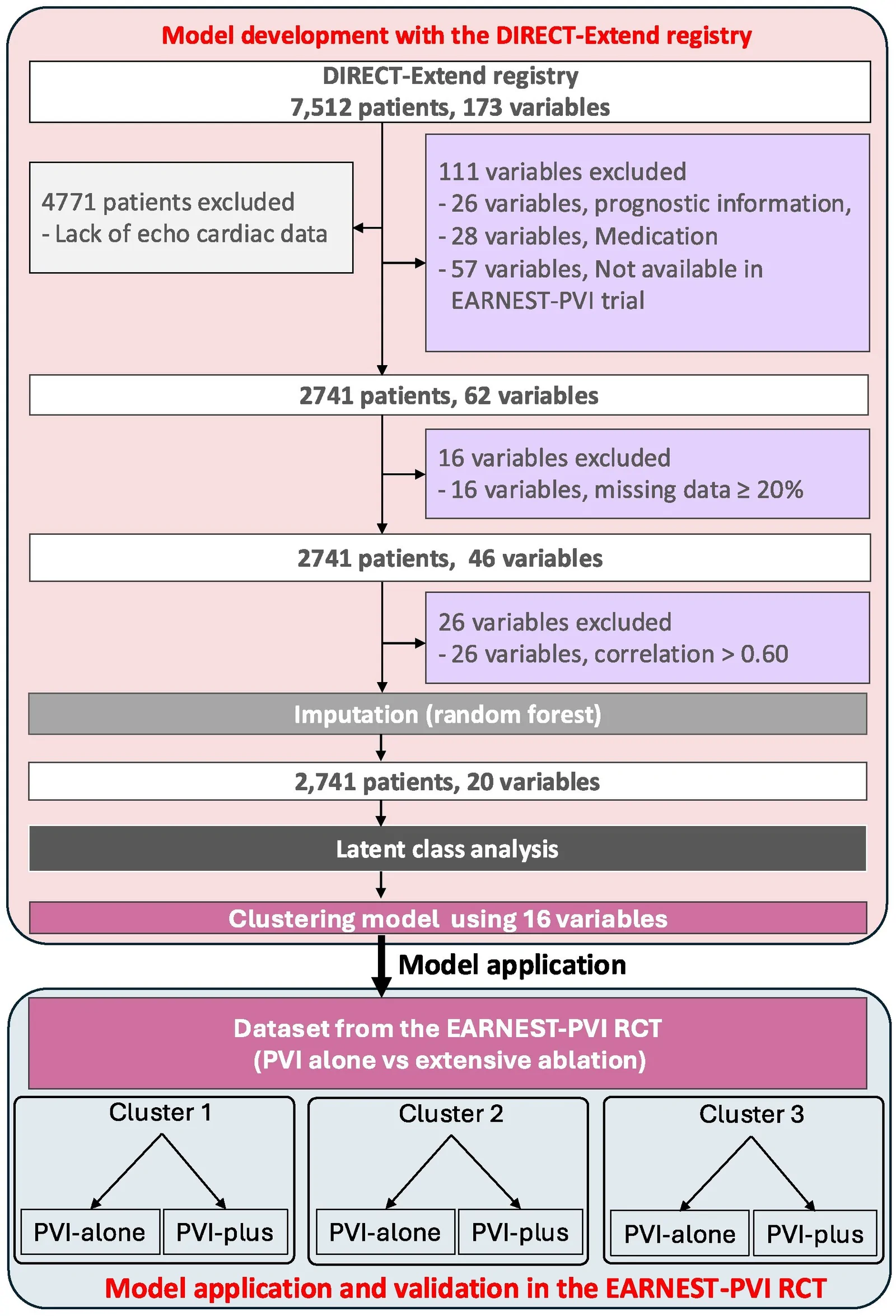
Study flow chart. Flow diagram of cohort assembly. Among 7512 patients initially enrolled across three AF registries (DIRECT, SAKURA-AF, Osaka University), those without echocardiographic data were excluded (including all SAKURA-AF patients due to lack of echo collection), yielding 2741 patients for latent class analysis (LCA). The final analysis set included clinical, laboratory, and echocardiographic variables with <20% missingness after imputation. PVI, pulmonary vein isolation.

**Figure 2:**
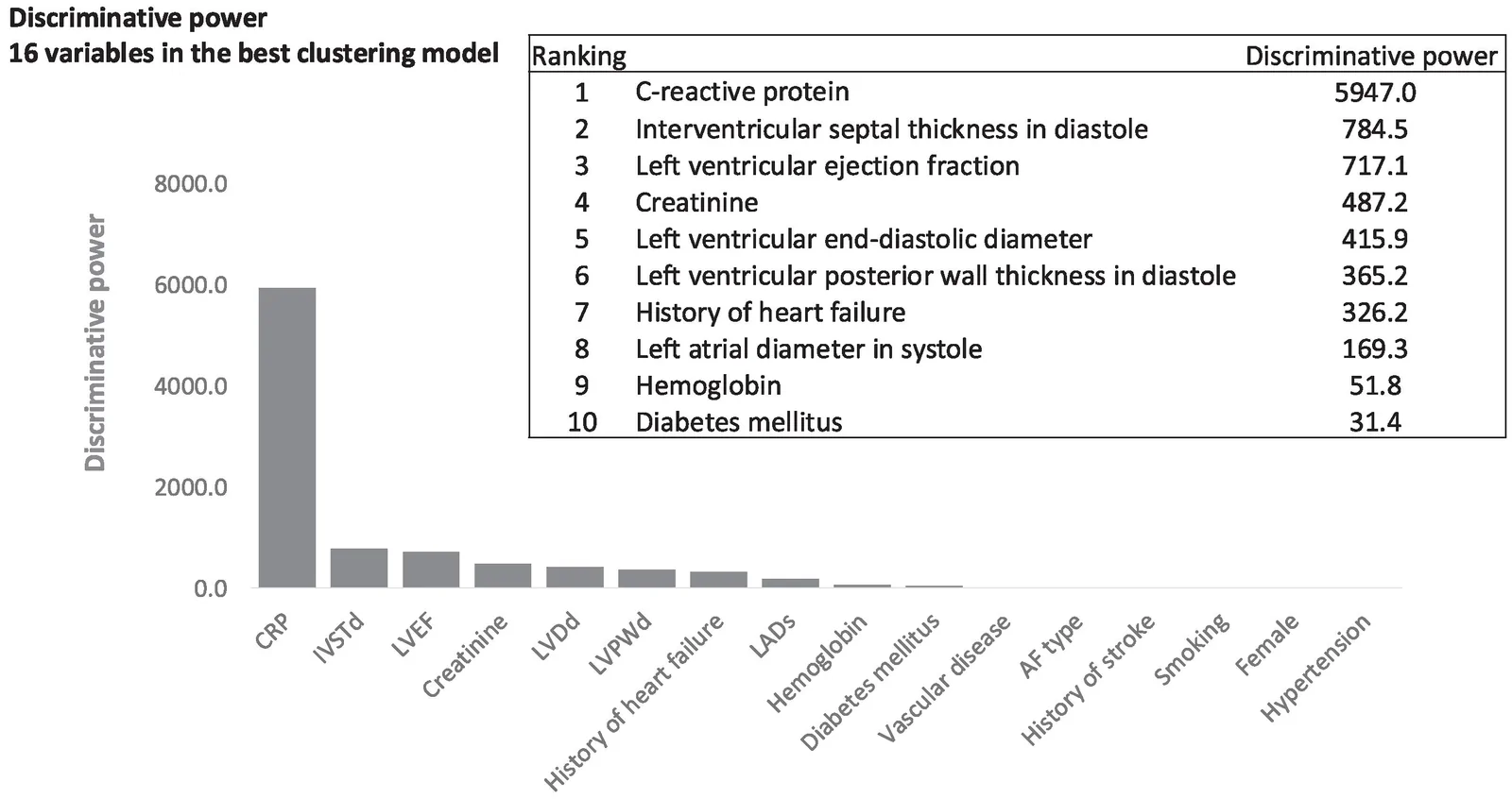
Discriminative power ranking. Discriminative power of the 16 variables selected by latent class analysis is shown as a bar graph. We computed the discriminative power of each variable as the logarithm of the ratio between the probability that the variable is relevant for clustering and the probability that it is irrelevant for clustering. CRP, C-reactive protein; IVSTd, interventricular septum thickness in diastole; LVEF, LV EF; LVDd, LV diastolic dimension; LVPWTd, LV posterior wall thickness in diastole; LADs, left atrial diameter in systole.

### Phenotypes

All patients were subclassified into three phenotypes as follows: Phenotype 1, *N* = 1565 (57.1%); Phenotype 2, *N* = 981 (35.8%); Phenotype 3, *N* = 195 (7.1%). Patients’ characteristics across the three phenotypes are summarized in *Table 1*. Laboratory and echocardiographic data are shown in *Table 2*. Medication data are shown in Supplementary material online, *Table S6*. Relative differences in the clinical features among the three phenotypes are illustrated in *Figure 3*. We named these phenotypes ‘low comorbidity’, ‘cardiomyopathy and chronic kidney disease (CKD)’, and ‘inflammatory-enriched AF’. ‘Low comorbidity’ had higher EF and higher haemoglobin levels. ‘Cardiomyopathy and CKD’ had lower EF, higher prevalence of heart failure history, larger left atrial diameter, and poorer kidney function. Diabetes mellitus was more frequently observed in this group. ‘Inflammatory-enriched AF’ had higher CRP levels and LV hypertrophy.

**Figure 3:**
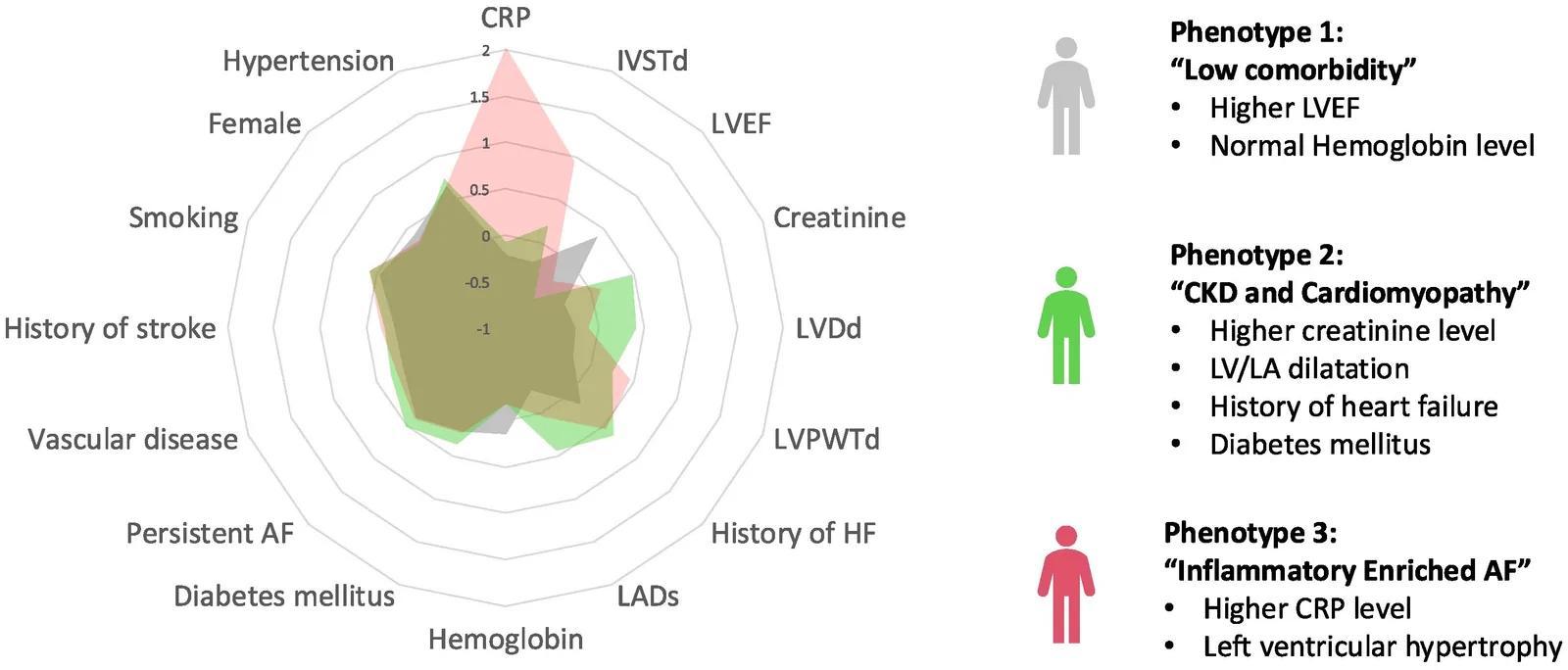
Clinical features in three phenotypes. Relative differences in the clinical features among the three phenotypes are illustrated in the radar chart. Phenotype 1 (‘low comorbidity/healthy AF’)—higher LVEF, higher haemoglobin, lower CRP; Phenotype 2 (‘cardiomyopathy & CKD’)—lower LVEF, larger LA size, impaired renal function, higher prevalence of diabetes, and heart failure history; Phenotype 3 (‘inflammatory-enriched AF’)—elevated CRP and increased ventricular wall thickness. Panel shows medians or proportions scaled to cohort distribution; darker colour indicates a higher relative value. Continuous variables are displayed as means of Z-standardized values, and categorical variables are displayed as proportions. For visualization purposes, AF type was grouped into paroxysmal AF and persistent/long-standing persistent AF, and smoking status was grouped into non-smoker and current or past smoker. CRP, C-reactive protein; IVSTd, interventricular septum thickness in diastole; LVDd, LV diastolic dimension; LVPWTd, LV posterior wall thickness in diastole; LVEF, LV EF; LADs, left atrial diameter in systole; CKD, chronic kidney disease; LV, left ventricle; LA, left atrium; HF, heart failure.

**Table 1:** Patient characteristics in the DIRECT-Extend registry

Variable	Phenotype 1	Phenotype 2	Phenotype 3	*P* value	Missing (%)
	*N* = 1565	*N* = 981	*N* = 195		
Age	72.0 (65.0, 78.0)	74.0 (65.0, 80.0)	75.0 (66.5, 81.5)	0.001	0
Female sex	616 (39.4)	288 (29.4)	63 (32.3)	<0.001	0
Body weight, kg	61.0 (52.0, 70.0)	62.0 (53.0, 72.0)	61.6 (50.8, 70.4)	0.019	0.3
Body mass index, kg/m^2^	22.9 (20.5, 25.9)	23.6 (20.5, 26.7)	23.4 (20.0, 26.7)	0.025	1.6
AF type				<0.001	0
Paroxysmal	1004 (64.2)	478 (48.7)	120 (61.5)		
Persistent	229 (14.6)	179 (18.2)	28 (14.4)		
Long-standing persistent	258 (16.5)	255 (26.0)	27 (13.8)		
Unknown	74 (4.7)	69 (7.0)	20 (10.3)		
Hypertension	1004 (64.2)	725 (73.9)	129 (66.2)	<0.001	0
Diabetes mellitus	324 (20.7)	356 (36.3)	43 (22.1)	<0.001	0
Dyslipidaemia	799 (51.1)	562 (57.3)	101 (51.8)	0.007	0
CAD	142 (9.1)	205 (20.9)	22 (11.3)	<0.001	0
History of stroke	303 (19.4)	293 (29.9)	66 (33.8)	<0.001	0
History of heart failure	240 (15.3)	622 (63.5)	105 (53.8)	<0.001	0
History of bleeding	192 (12.3)	199 (20.3)	26 (13.3)	<0.001	0.1
Vascular disease	301 (19.2)	322 (32.8)	52 (26.7)	<0.001	0
Smoking				<0.001	1.9
Never	805 (52.6)	410 (42.3)	78 (41.1)		
Past	564 (36.8)	437 (45.1)	70 (36.8)		
Current	162 (10.6)	122 (12.6)	42 (22.1)		
Liver dysfunction	344 (22.0)	258 (26.3)	59 (30.3)	0.005	0
History of malignant neoplasm	342 (21.9)	242 (24.7)	39 (20.0)	0.164	0
CHADS_2_ score	2.0 (1.0, 3.0)	3.0 (2.0, 4.0)	2.0 (1.0, 4.0)	<0.001	0.1
CHA_2_DS_2_-VASc score	3.0 (2.0, 4.0)	4.0 (3.0, 6.0)	4.0 (2.0, 6.0)	<0.001	0.1

Data with listwise deletion are expressed as median (interquartile range) or number (percentage). Liver dysfunction was defined according to the HAS-BLED score as the presence of chronic hepatic disease (e.g. cirrhosis) or biochemical evidence of significant hepatic derangement, such as bilirubin > 2 × the upper limit of normal (ULN) or AST/ALT/ALP > 3 × ULN.^34^

**Table 2:** Laboratory and echocardiographic data in the DIRECT-extend registry

Variable	Phenotype 1	Phenotype 2	Phenotype 3	*P* value	Missing (%)
	*N* = 1565	*N* = 981	*N* = 195		
Laboratory data					
Haemoglobin, g/dL	13.6 (12.5, 14.9)	12.9 (11.3, 14.6)	13.1 (11.5, 14.3)	<0.001	2.7
Platelets, 10^3/µL	191.0 (160.0, 227.0)	190.0 (153.0, 233.0)	209.0 (164.0, 254.0)	0.004	3.6
Creatinine, mg/dL	0.8 (0.7, 0.9)	1.0 (0.8, 1.2)	0.9 (0.7, 1.1)	<0.001	2.7
CRP, mg/L	0.0 (0.0, 0.1)	0.1 (0.0, 0.3)	1.3 (0.9, 2.4)	<0.001	7
BNP, pg/mL	90.7 (40.4, 181.7)	175.6 (91.8, 371.0)	267.9 (108.5, 516.0)	<0.001	59.1
NT-proBNP, pg/mL	395.4 (138.8, 935.6)	1270.0 (630.6, 2771.0)	1733.5 (1062.0, 4342.0)	<0.001	50.4
Albumin, g/dL	4.1 (3.8, 4.3)	3.8 (3.4, 4.1)	3.3 (2.9, 3.9)	<0.001	39.1
PT-INR	1.1 (1.0, 1.4)	1.2 (1.1, 1.4)	1.1 (1.0, 1.3)	<0.001	22.1
Total bilirubin, mg/dL	0.7 (0.5, 0.9)	0.7 (0.5, 1.0)	0.7 (0.5, 1.0)	0.974	8.4
AST, IU/L	24.0 (20.0, 31.0)	25.0 (19.0, 32.0)	26.0 (19.0, 37.0)	0.109	3.2
ALT, IU/L	19.0 (14.0, 27.0)	20.0 (13.0, 30.0)	20.0 (13.0, 33.0)	0.721	3.1
Echo cardiac data					
LVDd, mm	47.0 (44.0, 51.0)	51.0 (46.0, 57.0)	48.0 (43.0, 52.0)	<0.001	0
LVDs, mm	29.0 (27.0, 32.0)	35.0 (29.0, 44.0)	31.0 (27.0, 37.8)	<0.001	0.1
IVSTd, mm	8.0 (8.0, 9.0)	9.0 (8.0, 11.0)	9.3 (8.0, 12.0)	<0.001	0.3
LVPWTd, mm	8.0 (8.0, 9.0)	9.0 (8.0, 11.0)	9.0 (8.0, 10.0)	<0.001	0.4
LVEF, %	67.0 (62.0, 72.0)	58.0 (42.0, 68.0)	62.0 (48.0, 69.0)	<0.001	0
LADs, mm	43.0 (39.0, 48.0)	48.0 (44.0, 54.0)	46.0 (40.0, 51.0)	<0.001	0

Data with listwise deletion are expressed as median (interquartile range) or number (percentage).

LVDd, LV diastolic dimension; LVDs, LV systolic dimension; IVSTd, interventricular septum thickness in diastole; LVPWTd, LV posterior wall thickness in diastole; LVEF, LV EF; LADs, left atrial dimension in systole; TRPG, tricuspid regurgitation pressure gradient; TAPSE, tricuspid annular plane systolic excursion.

### Association of phenotypes with clinical outcomes

The median follow-up duration was 600 (121–1565) days. Kaplan–Meier analysis showed a significant difference in the primary endpoint between the phenotypes (*Figure 4*). Patients in Phenotype 3 had the worst prognosis, while Phenotype 1 showed the best prognosis. Secondary outcomes are shown in Supplementary material online, *Figure S3*. All-cause mortality, major bleeding, clinically relevant non-major bleeding, and any bleeding were significantly worse in Phenotype 3 compared to the other phenotypes. Heart failure admission was significantly worse in Phenotype 2. In contrast, no significant differences in the risks of myocardial infarction, any stroke, haemorrhagic stroke, or ischaemic stroke were observed across phenotypes.

**Figure 4:**
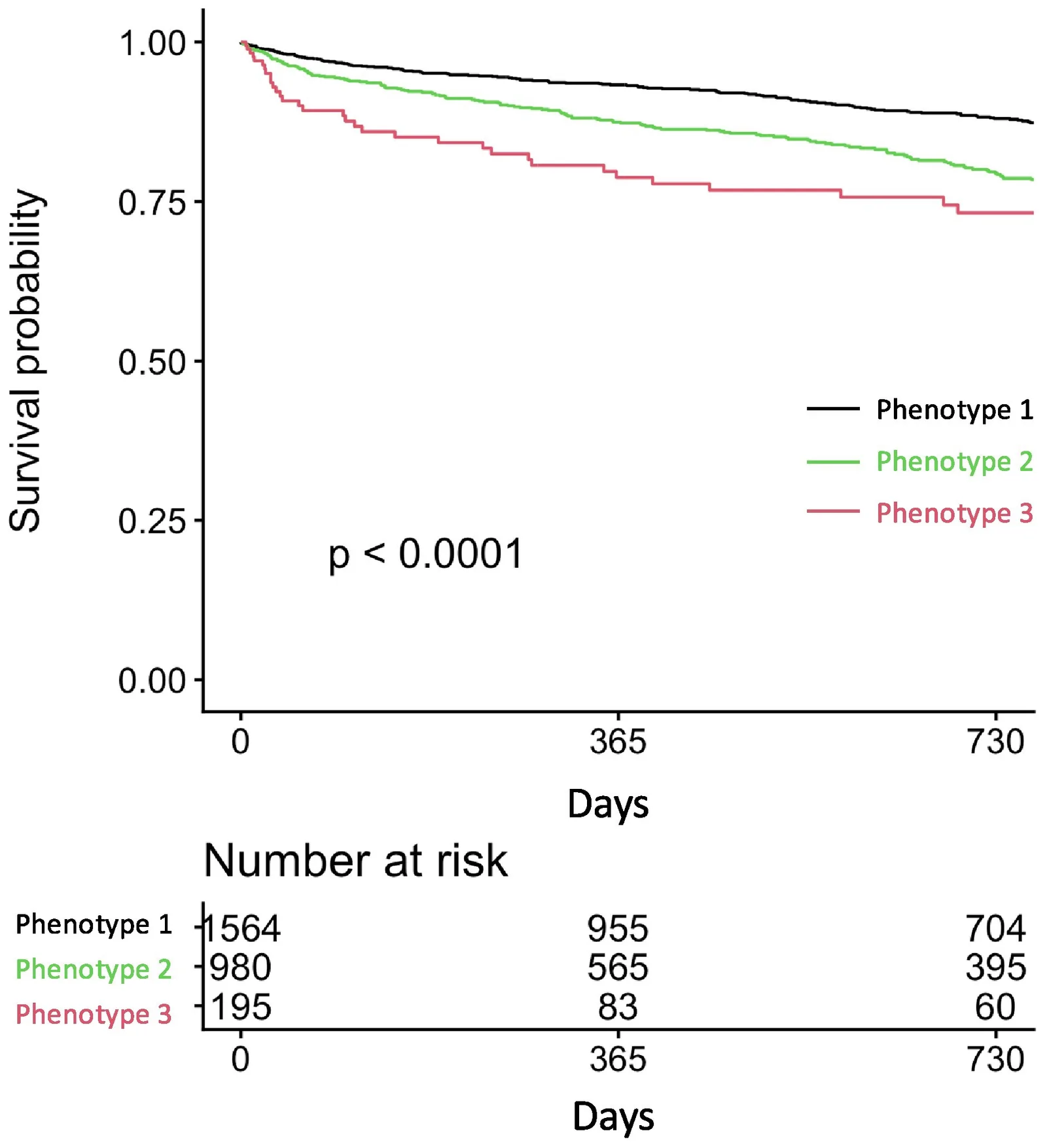
Clinical outcomes in the DIRECT-extend registry. Survival analysis was conducted with Kaplan–Meier methods for a composite of all-cause death, acute myocardial infarction, any stroke, and major bleeding according to the International Society on Thrombosis and Haemostasis criteria.

### Application of phenotyping model to the EARNEST-PVI trial

Application of the previously established phenotyping model to the EARNEST-PVI cohort classified patients into three phenotypes. Phenotype 1, *N* = 328 (66.0%); Phenotype 2, *N* = 139 (28.0%); and Phenotype 3, *N* = 30 (6.0%). Patients’ characteristics across the three phenotypes are summarized in Supplementary material online, *Table S7*. The distribution of phenotypes and their clinical characteristics were largely consistent with those observed in the DIRECT-Extend cohort, supporting the external validity of the phenotyping model. Briefly, Phenotype 1 showed the lowest burden of comorbidities and inflammation. Phenotype 2 was characterized by a high prevalence of diabetes and a history of heart failure, along with dilatation of the LV end-diastolic diameter and the left atrial diameter and modest renal impairment. Phenotype 3 exhibited an inflammation-dominant profile, with elevated CRP levels. The procedural details of extensive ablation in the PVI-plus group are summarized in Supplementary material online, *Table S8*. The median follow-up duration was 1330 (1099–1500) days. The recurrence rate was significantly lower in the PVI-plus group compared to the PVI-alone group in phenotype 1 [PVI-alone 78/168 (46.4%) vs. PVI-plus 54/160 (33.8%), log-rank *P* = 0.013; hazard ratio (HR) 0.646 (0.456–0.914), *P* = 0.013]. In contrast, there was no significant difference in recurrence rates between the PVI-alone and PVI-plus groups in Phenotypes 2 and 3 [Phenotype 2: PVI-alone 23/65 (35.4%) vs. PVI-plus 28/74 (37.8%), log-rank *P* = 0.79; HR 1.080 (0.622–1.880), *P* = 0.780; Phenotype 3: PVI-alone 5/16 (31.2%) vs. PVI-plus 2/14 (14.3%), log-rank *P* = 0.53; HR 0.478 (0.009–2.470), *P* = 0.378] (*Figure 5*). A formal test for interaction between treatment strategy and phenotype was performed using a Cox proportional hazards model including an interaction term. Although the Kaplan–Meier curves suggested a numerically greater separation between the PVI-plus group and the PVI-alone group in Phenotype 1 compared with the other phenotypes, the interaction was not statistically significant (*P* for interaction = 0.229).

**Figure 5:**
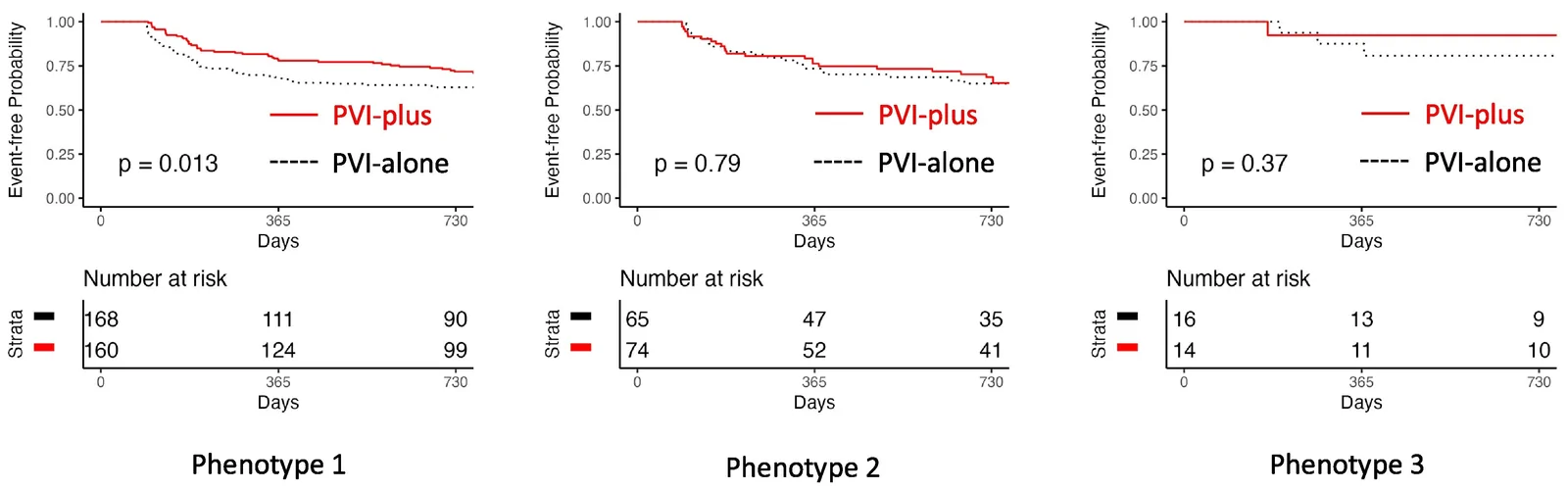
Ablation strategy outcomes by phenotype in the EARNEST-PVI trial. Freedom from atrial arrhythmia recurrence after ablation, stratified by phenotype and randomized strategy: pulmonary-vein isolation alone (PVI-alone) vs. PVI with extensive ablation (PVI-plus). PVI, pulmonary vein isolation.

## Discussion

The main findings of the present study were summarized as follows: (1) We demonstrated that the machine-learning-based unsupervised clustering approach successfully identified phenotypes in a real-world East Asian cohort of AF patients; (2) The three phenotypes identified in the current study had distinct baseline characteristics and clinical outcomes; (3) When externally applied to the EARNEST-PVI randomized trial, the phenotyping model demonstrated good generalizability, classifying patients into comparable subgroups with similar characteristics; and (4) Extensive ablation strategy was associated with a significantly lower recurrence rate compared with PVI alone in patients classified as Phenotype 1. To the best of our knowledge, this is the first study in which phenotyping-based stratification was applied to an existing prospective randomized trial to assess differences in treatment response to the AF ablation strategy.

### Features of phenotypes

Several prior studies have shown that AF is not a single disease entity but consists of distinct clinical phenotypes. In the CABANA (Catheter ABlation vs ANtiarrhythmic Drug Therapy for Atrial Fibrillation) trial (*N* = 2205), cluster analysis identified three groups: (1) a relatively healthy phenotype with predominantly paroxysmal AF and few comorbidities, (2) a younger, male, higher-body mass index group with more persistent AF but otherwise low risk, and (3) an older multimorbid group with impaired renal function and the highest event rates.^17^ In an Italian single-centre AF ablation cohort (*N* = 209), three clinical phenotypes were identified: (1) a low-risk group with smaller atria and generally favourable post-ablation outcomes, (2) an intermediate group characterized by atrial enlargement that frequently required more extensive ablation, and (3) a high-risk group distinguished by heart failure, multiple comorbidities, and elevated inflammatory biomarkers such as CRP, which corresponded to the highest rates of AF recurrence.^18^

Our study showed that patients with AF could be stratified into three distinct clinical phenotypes based on systemic and cardiac profiles (*Figure 3*). Phenotype 1 represents a relatively healthy population with fewer comorbidities and favourable clinical outcomes, corresponding to a lower-risk subgroup of AF patients. Phenotype 2 includes patients with impaired renal function, diabetes mellitus, and cardiomyopathic features as indicated by reduced LV EF and enlarged left atrial size. This group was characterized by advanced atrial and ventricular remodelling and showed a higher incidence of heart failure-related outcomes. Phenotype 3 was defined by elevated CRP levels, indicative of systemic inflammation, and by increased LV wall thickness. Patients in this phenotype had the poorest prognosis, with higher rates of death and bleeding events.

A common finding across previous phenotyping studies and our analysis is that systemic inflammation represents a key determinant of AF pathophysiology. Consistent with this concept, several studies have demonstrated that inflammation contributes to adverse outcomes.^19–21^ Systemic inflammation has been shown to promote endothelial dysfunction and vascular fragility, potentially increasing susceptibility to bleeding and vascular complications.^22,23^ Recognizing such an inflammation-related phenotype may help identify patients at higher risk for the adverse events and guide more individualized management strategies. Furthermore, consistent with prior phenotyping studies, our analysis also identified a subgroup analogous to the comorbidity-burdened cluster—represented by Phenotype 2—characterized by CKD, heart failure, and structural cardiac remodelling. In contrast, unlike prior clustering analyses in which obesity and age differences played a dominant role in phenotype separation, these factors were less pronounced in our cohort. This discrepancy may be attributable to differences in cohort characteristics, including racial composition, clinical background, and patient selection criteria.

An important strength of our study is the inclusion of echocardiographic data reflecting cardiac structural remodelling, which have rarely been incorporated in previous AF phenotyping studies.^6,17,24^ By integrating these cardiac morphological variables, our analysis provided additional characterization of AF patients and supported phenotypic stratification in relation to clinical outcomes. Interestingly, although Phenotype 2 was characterized by a higher burden of traditional comorbidities such as diabetes mellitus, hypertension, CKD, and heart failure, clinical outcomes were worse in Phenotype 3, which was characterized by features such as higher CRP levels and increased ventricular wall thickness. This finding suggests that prognosis may not be fully explained by comorbidity burden alone and highlights the potential importance of other factors, such as systemic inflammation and echocardiographic information, captured by the present phenotyping approach. While the individual variables incorporated into the clustering model are established prognostic factors, the novelty of the present approach lies in integrating these complementary clinical, laboratory, and echocardiographic dimensions into clinically meaningful multidimensional phenotypes, rather than evaluating each variable in isolation. This integrated approach may provide additional insights into patient heterogeneity that are not readily apparent when considering individual risk factors separately.

### Efficacy of extensive ablation strategy across three phenotypes

The most important advantage of our study over prior work is that we went beyond clustering alone by implementing our approach in an existing randomized controlled trial dataset and demonstrating differences in the effectiveness of ablation strategies. In this study, an extensive ablation strategy was associated with a reduced recurrence rate in Phenotype 1. In contrast, there was no statistically significant difference between PVI alone and PVI-plus in Phenotypes 2 and 3. This finding was somewhat unexpected in light of our previously reported subgroup analyses within the EARNEST-PVI trial, which suggested a preferential benefit of extensive ablation beyond PVI alone in patients with older age, mitral regurgitation, or higher DR-FLASH scores, all of which are thought to be associated with more advanced atrial substrate.^25–27^ Contrary to expectations derived from prior subgroup analyses, our phenotype-based analysis demonstrated that extensive ablation strategy was associated with a reduction in AF recurrence in Phenotype 1, a group characterized by a low comorbidity burden, rather than in phenotypes defined by markers traditionally associated with advanced atrial substrate.

The exploratory findings regarding treatment responses to extensive ablation strategy across phenotypes may be better interpreted by considering qualitative differences in the nature of atrial remodelling, rather than its extent alone. Specifically, atrial remodelling may differ between localized anatomical or mechanical processes and diffuse systemic or inflammatory processes associated with greater electrophysiological complexity. Phenotype 1 included a substantial proportion of patients with moderate or greater mitral regurgitation, which is consistent with our prior subgroup analyses from the EARNEST-PVI trial showing a favourable response to extensive ablation strategy in such patients.^27^ Prior studies have shown that chronic atrial stretch in mitral regurgitation is associated with atrial remodelling, particularly in the posterior left atrium.^28^ Given that the posterior left atrium is frequently targeted as part of additional lesion sets in extensive ablation strategies, these regionally distributed abnormalities may be more effectively modified by such approaches. Although speculative, Phenotype 1 may be characterized by a more localized or anatomically and mechanically determined distribution of arrhythmogenic substrate, which may be more amenable to modification by extensive ablation strategy. By contrast, in Phenotype 2, prior studies have reported that systemic conditions such as CKD and diabetes mellitus promote diffuse atrial remodelling.^29,30^ Consistent with this concept, a subgroup analysis from the EARNEST-PVI trial demonstrated that patients with diabetes mellitus did not derive significant benefit from an extensive ablation strategy compared with PVI-alone ablation, further supporting the notion that systemic metabolic disease attenuates the therapeutic effect of extensive ablation.^31^ Phenotype 3 was characterized by elevated CRP levels, which may reflect a greater contribution of inflammatory processes to atrial remodelling. In this phenotype, no statistically significant difference in recurrence was observed between the PVI-plus group and the PVI-alone group. However, given the relatively small sample size of this phenotype, these findings should be regarded as descriptive exploratory observations and interpreted with considerable caution. Previous studies have shown that high CRP levels are independent predictors of late AF recurrence after ablation.^32^ Inflammation in the left atrium also reportedly plays a critical role in the generation and progression of AF substrate.^32,33^ Persistent inflammatory activity may represent one possible explanation for the limited effectiveness of substrate modification observed in this phenotype, although this hypothesis requires further investigation. These findings collectively indicate that the additional benefit of empiric extensive ablation strategy may be limited among populations such as Phenotypes 2 and 3 in which atrial remodelling is primarily driven by systemic comorbidities or ongoing inflammatory processes. In such settings, achieving robust substrate modification may be challenging, potentially resulting in incomplete lesion formation and conduction gaps that facilitate reentrant circuits and AF recurrence. Taken together, these observations underscore the variability in treatment response to extensive ablation strategy across patient phenotypes.

From a clinical perspective, these findings highlight that phenotypic stratification of AF patients allows not only for prognostic discrimination but also for a more individualized approach to catheter ablation therapy. Given the substantial heterogeneity in comorbidities and disease severity among patients with AF, uniformly applying empiric extensive ablation to all patients may not be optimal. Instead, multidimensional phenotypic stratification may eventually help guide patient selection for empiric extensive ablation rather than adopting a one-size-fits-all approach. Our findings highlight the potential of an ‘AI-supported ablation strategy’, which may eventually support individualized treatment recommendations. Although experienced clinicians can often estimate patient risk based on clinical presentation, a multidimensional phenotyping approach may provide a structured and reproducible framework to assist clinical judgment. By integrating clinical and echocardiographic information, this approach may help characterize patient heterogeneity and identify subgroups with differential responses to ablation strategies. Future studies should validate these findings in prospective randomized settings and develop clinically applicable phenotyping tools, such as user-friendly machine learning–based classification models, to facilitate implementation in routine clinical practice.

### Limitations

Several limitations must be acknowledged. First, this study is a multicentre East Asian AF registry, which restricts the generalizability of the findings to other racial groups. Second, the relatively small sample size may have underpowered the analysis to detect the effect of extensive ablation strategy in the EARNEST-PVI trial, particularly in Phenotype 3 (*N* = 30), and potentially also in Phenotype 2 (*N* = 139). Moreover, the application of the phenotyping model to the EARNEST-PVI trial was performed as a *post hoc* analysis. Therefore, the findings regarding differential treatment effects across phenotypes should be interpreted with caution and be considered exploratory and hypothesis-generating. Third, phenotype assignment relied on baseline clinical variables; incorporation of imaging, electro-anatomical mapping, or biomarker data may refine classification. Prospective randomized trials stratified by predefined phenotypes are now warranted to confirm that phenotype-guided ablation improves long-term outcomes. Fourth, it should be noted that the DIRECT-Extend registry used to derive the phenotyping model included both paroxysmal and persistent AF, whereas the EARNEST-PVI trial exclusively enrolled patients with persistent AF. Importantly, AF type itself was not a major driver of cluster formation in the present analysis. This finding suggests that the identified phenotypes were determined primarily by systemic and structural cardiac characteristics rather than by AF duration-based classifications. Accordingly, although the evaluation of ablation response was performed in a cohort of persistent AF patients, the phenotyping framework may capture broader disease biology beyond conventional AF types. Nevertheless, whether similar phenotype–treatment interactions exist in patients with paroxysmal AF warrants further investigation. Fifth, a substantial proportion of patients were excluded due to missing echocardiographic data. Among 7512 patients in the DIRECT-Extend registry, only 2741 were included in the present analysis. As echocardiographic parameters were essential for defining structural phenotypes, patients without such data could not be included. Patients with available echocardiographic data may not be fully representative of the overall AF population, as they may systematically differ in their clinical characteristics and outcomes from those without such data. This introduces a risk of selection bias and may limit the external validity and generalizability of our findings. However, several echocardiographic variables showed high discriminative power in the LCA, suggesting that echocardiographic assessment provides important information for phenotypic classification beyond clinical variables alone. Thus, incorporating echocardiographic information represents a strength of the present study by enabling a more comprehensive characterization of AF heterogeneity. Nevertheless, whether similar phenotypes can be reproduced using clinical variables alone remains uncertain and warrants further investigation. Sixth, blinding of outcome assessment was not performed due to the observational nature of the study. Seventh, clinical events were not adjudicated by a clinical events committee, and event classification solely relied on the available clinical records. Eighth, external validation was not performed; therefore, comparison between development and evaluation datasets was not applicable. Ninth, the extensive ablation strategy evaluated in the EARNEST-PVI trial comprised a heterogeneous combination of lesion sets rather than a single standardized intervention. Therefore, the present findings should be interpreted as reflecting the overall extensive ablation strategy, and future studies are needed to determine whether specific lesion sets have differential efficacy across patient phenotypes. Finally, the phenotypes were derived using an unsupervised clustering approach based solely on baseline clinical characteristics, without incorporating outcome variables. While the phenotypes identified in the DIRECT-Extend registry were associated with hard clinical outcomes, supporting their prognostic relevance, the evaluation in the EARNEST-PVI trial was based on arrhythmia recurrence, a distinct rhythm-related endpoint. Therefore, further validation of these phenotypes in relation to both hard clinical outcomes and rhythm-related outcomes is warranted in future studies.

## Conclusions

LCA revealed distinct clinical phenotypes in patients with AF and demonstrated their associations with different prognoses. Notably, these phenotypes may be associated with heterogeneous treatment responses. Our findings suggest that phenotyping may not only improve risk stratification but also help tailor ablation strategies, potentially contributing to more personalized AF management.

## Supplementary Material

oeag135_Supplementary_Data

## Data Availability

Our study data will not be made available to other researchers for purposes of reproducing the results because of institutional review board restrictions. Analytical code is available from the corresponding author upon reasonable request.
