# Supplementary material for: Phenotypic clustering of atrial fibrillation patients using latent class analysis and its potential utility for ablation strategy: insights from the DIRECT-Extend and EARNEST-PVI studies
Source: Eur Heart J Open. 2026 Sep 8;6(5):oeag135. doi: 10.1093/ehjopen/oeag135 (PMC13643170; doi:10.1093/ehjopen/oeag135)
Supplement: Supplementary file 1 [file oeag135_supplementary_data.docx]

**Supplementary data**

**Phenotypic Clustering of Atrial Fibrillation Patients Using Latent Class Analysis and Its Potential Utility for Ablation Strategy: Insights from the DIRECT-Extend and EARNEST-PVI Studies**

**Supplemental Method**

**Supplementary Tables**

Table S1. Variables included latent class analysis

Table S2. TRIPOD+AI Checklist for Reporting of the Present Study

Table S3. Compliance with the EHRA AI checklist for reporting artificial intelligence research in electrophysiology

Table S4. Baseline characteristics and outcomes of included vs. excluded patients based on availability of echocardiographic data

Table S5. Baseline characteristics of phenotypes identified without prior imputation (sensitivity analysis)

Table S6. Medications in the DIRECT-Extend registry

Table S7. Patients’ characteristics across the 3 phenotypes in the EARNEST-PVI trial

Table S8. Extensive Ablation in EARNEST-PVI trial

**Supplementary Figures**

Figure S1. Bayesian information criterion

Figure S2. Discriminative power ranking

Figure S3. Probabilities of misclassification

Figure S4. Secondary outcomes in the DIRECT-Extend registry

**Supplemental Methods**

**Reproducibility and model stability**

To ensure reproducibility, fixed random seeds were applied in both the imputation process and the clustering analysis. Specifically, the imputation procedure was performed with a predefined random seed, and the clustering algorithm was also initialized with a fixed seed. In addition, the stability of the clustering results was assessed by repeating the analysis with different initializations.

**Assessment of heterogeneity across phenotypes**

Although this study was not specifically designed to investigate health inequalities, potential differences across sociodemographic groups such as age and sex were considered in the analysis. To characterize heterogeneity between identified phenotypes, differences in clinical characteristics and outcomes across clusters were systematically evaluated.

**Fairness considerations**

No formal fairness assessment was performed, as this study did not involve predictive modeling for clinical decision-making.

**Patient and public involvement**

Patients or the public were not involved in the design, conduct, reporting, or dissemination of this research due to the retrospective nature of the study.

**Intended users of the model**

The phenotyping model is intended for use by clinicians with basic knowledge of clinical data interpretation.

**Table S1. Variables included latent class analysis**

| **Number** | **Features** | **Type of data** | **Unit*/Options†** |
| --- | --- | --- | --- |
| 1 | Age | Continuous | years |
| 2 | Female sex | Nominal | yes/no |
| 3 | Body Mass Index | Continuous | kg/m² |
| 4 | Type of Atrial Fibrillation | Nominal | paroxysmal/persistent/long-standing persistent |
| 5 | History of Stroke or Transient Ischemic Attack | Nominal | yes/no |
| 6 | Hypertension | Nominal | yes/no |
| 7 | Diabetes Mellitus | Nominal | yes/no |
| 8 | Dyslipidemia | Nominal | yes/no |
| 9 | History of Heart Failure | Nominal | yes/no |
| 10 | Vascular Disease | Nominal | never/past/current |
| 11 | Smoking History | Nominal | yes/no |
| 12 | Liver Dysfunction | Nominal | yes/no |
| 13 | Hemoglobin | Continuous | g/dL |
| 14 | Creatinine | Continuous | mg/dL |
| 15 | C-Reactive Protein | Continuous | mg/dL |
| 16 | Left Ventricular End-Diastolic Diameter | Continuous | mm |
| 17 | Interventricular Septal Thickness in Diastole | Continuous | mm |
| 18 | Left Ventricular Posterior Wall Thickness in Diastole | Continuous | mm |
| 19 | Left Ventricular Ejection Fraction | Continuous | % |
| 20 | Left Atrial Diameter in Systole | Continuous | mm |

*Unit for continuous value. †Options for nominal or ordinal value

**Table S2. TRIPOD+AI Checklist for Reporting of the Present Study**

| **Section** | **Item** | **Description** | **Location in manuscript** |
| --- | --- | --- | --- |
| Title | 1 | Identify study as prediction model | Title page |
| Abstract | 2 | Structured abstract | Abstract |
| Introduction | 3a | Background & rationale | Introduction |
| Introduction | 3b | Target population & intended use | Introduction |
| Introduction | 3c | Health inequalities | Statistical Analysis |
| Introduction | 4 | Objectives | Introduction end |
| Methods | 5a | Data source | Methods |
| Methods | 5b | Study dates | Methods |
| Methods | 6a | Study setting | Methods |
| Methods | 6b | Eligibility criteria | Methods |
| Methods | 6c | Treatment handling | Methods |
| Methods | 7 | Data preprocessing | Statistical Analysis |
| Methods | 8a | Outcome definition | Clinical Outcomes |
| Methods | 8b | Outcome assessor | Clinical Outcomes/Limitations |
| Methods | 8c | Blinding | Clinical Outcomes/Limitations |
| Methods | 9a | Predictor selection | Statistical Analysis |
| Methods | 9b | Predictor definition | Clinical variables |
| Methods | 9c | Predictor assessor | Clinical variables |
| Methods | 10 | Sample size | Statistical Analysis |
| Methods | 11 | Missing data | Statistical Analysis |
| Methods | 12a | Data usage | Statistical Analysis |
| Methods | 12b | Predictor handling | Statistical Analysis |
| Methods | 12c | Model specification | Statistical Analysis |
| Methods | 12d | Heterogeneity | Statistical Analysis |
| Methods | 12e | Model performance | Statistical Analysis |
| Methods | 12f | Model updating | Not applicable |
| Methods | 12g | Prediction calculation | Statistical Analysis |
| Methods | 13 | Class imbalance | Statistical Analysis |
| Methods | 14 | Fairness | Statistical Analysis |
| Methods | 15 | Model output | Statistical Analysis |
| Methods | 16 | Training vs evaluation differences | Methods |
| Methods | 17 | Ethical approval | Methods |
| Open science | 18a | Funding | Funding section |
| Open science | 18b | Conflicts of interest | Disclosures |
| Open science | 18c | Protocol | Methods |
| Open science | 18d | Registration | Methods |
| Open science | 18e | Data sharing | Data availability |
| Open science | 18f | Code sharing | Data availability |
| Methods | 19 | Patient & public involvement | Supplemental Method |
| Results | 20a | Flow of participants | Figure / Results |
| Results | 20b | Participant characteristics | Table 1 |
| Results | 20c | Comparison dev vs eval | Not applicable |
| Results | 21 | Sample size per analysis | Results |
| Results | 22 | Model specification details | Results |
| Results | 23a | Model performance | Results |
| Results | 23b | Performance heterogeneity | Results |
| Results | 24 | Model updating results | Not applicable |
| Discussion | 25 | Interpretation | Discussion |
| Discussion | 26 | Limitations | Discussion |
| Discussion | 27a | Handling input data issues | Not described |
| Discussion | 27b | User requirements | Not described |
| Discussion | 27c | Future directions | Discussion |

**Table S3. Compliance with the EHRA AI checklist for reporting artificial intelligence research in electrophysiology**

| Item | Checklist item | Description in the present study | Location in manuscript |
| --- | --- | --- | --- |
| Title | Identification of AI/ML | Machine-learning-based unsupervised clustering (latent class analysis) is clearly stated | Title / Methods |
| 1 | Intended clinical use | To identify clinically meaningful AF phenotypes and guide ablation strategy | Introduction |
| 2 | Clinical benefit | Potential improvement in risk stratification and treatment selection beyond conventional approaches | Introduction / Discussion |
| 3 | Data collection | Data derived from the DIRECT-Extend registry | Methods |
| 4 | Source of data | Multicenter observational registry data | Methods |
| 5 | Development dataset | DIRECT-Extend cohort used for model development | Methods |
| 6 | Participants | Inclusion/exclusion criteria described; baseline characteristics provided | Methods / Table 1 |
| 7 | Comparator | Not applicable (unsupervised learning without predefined labels) | Methods |
| 8 | Testing dataset | External validation performed in EARNEST-PVI cohort | Methods |
| 9 | Sample size | Determined by available registry population; sufficiently large for clustering | Methods |
| 10 | Outcome | Clinical outcomes including AF recurrence and major adverse events | Methods |
| 11 | Data type | Clinical variables, laboratory data, echocardiographic parameters | Methods |
| 12 | Data preparation | Variables with ≥20% missingness excluded; imputation performed using random forest method | Methods |
| 13 | Balanced groups | Not applicable for unsupervised clustering | Methods |
| 14 | Data issues | Missing data handled via imputation; non-available variables excluded | Methods |
| 15 | Feature selection | Variable selection performed based on clinical relevance and availability across datasets | Methods |
| 16 | Legal framework | Not applicable (research use only) | – |
| 17 | Explainability | Cluster characteristics interpreted clinically using baseline variables | Results / Discussion |
| 18 | Ethical approval | Approved by institutional review boards; informed consent obtained | Methods |
| 19 | Fairness | Broad patient population included; no specific subgroup exclusion beyond study criteria | Methods |
| 20 | Data/code availability | Data not publicly available due to privacy restrictions; available upon request | Methods |
| 21 | Trial registration | Parent registries registered; details provided | Methods |
| 22 | Participants (results) | Baseline characteristics of clusters described | Results |
| 23 | Training performance | Model fit assessed using statistical criteria (e.g., BIC) | Methods / Results |
| 24 | Internal validation | Stability assessed within derivation cohort | Methods |
| 25 | External validation | Clusters applied to independent cohort (EARNEST-PVI) | Methods / Results |
| 26 | Model performance | Association of clusters with outcomes evaluated using survival analysis | Results |
| 27 | Performance errors | Limitations of clustering and misclassification discussed | Discussion |
| 28 | Comparison with conventional methods | Compared with conventional clinical stratification approaches | Discussion |
| 29 | Generalizability | External validation supports applicability across populations | Discussion |
| Conclusion | Supported by data | Conclusions consistent with results and exploratory nature acknowledged | Discussion / Conclusion |

**Table S4. Baseline characteristics and outcomes of included vs. excluded patients based on availability of echocardiographic data**

| **Variable** | **Included cohort (echocardiographic data available)** | **Excluded cohort (echocardiographic data unavailable)** | **P value** | **Missing (%)** |
| --- | --- | --- | --- | --- |
|  | **N = 2741** | **N = 4771** |  |  |
| Age | 73.0 [65.0, 79.0] | 72.00 [66.0, 79.0] | 0.877 | 0 |
| Female sex | 967(35.3) | 1364(28.6) | <0.001 | 0 |
| Body weight, Kg | 61.35 [52.00, 70.60] | 63.00 [54.00, 71.15] | <0.001 | 0.4 |
| Body mass index, Kg/m2 | 23.23 [20.47, 26.28] | 23.66 [21.34, 26.17] | <0.001 | 1.5 |
| AF type |  |  | <0.001 | 0 |
| Paroxysmal | 1602(58.4) | 2151(45.1) |  |  |
| Persistent or long persistent | 1062(38.7) | 2552(53.5) |  |  |
| Unknown | 77(2.8) | 68(1.4) |  |  |
| Hypertension | 1858(67.8) | 3334(69.9) | 0.062 | 0 |
| Diabetes mellitus | 723(26.4) | 1119(23.5) | 0.005 | 0 |
| Dyslipidemia | 1462(53.4) | 2089(43.8) | <0.001 | 0 |
| Coronary artery disease | 369(13.5) | 459(9.6) | <0.001 | 0 |
| History of stroke | 662(24.2) | 711(14.9) | <0.001 | 0 |
| History of heart failure | 967(35.3) | 1106(23.2) | <0.001 | 0 |
| History of bleeding | 417(15.2) | 255(5.3) | <0.001 | 0.1 |
| Vascular disease | 675(24.6) | 714(15.0) | <0.001 | 0 |
| Smoking |  |  | 0.315 | 1.3 |
| Never | 1293(48.1) | 2317(49.1) |  |  |
| Past | 1071(39.8) | 1799(38.1) |  |  |
| Current | 326(12.1) | 605(12.8) |  |  |
| Liver dysfunction | 661(24.1) | 344(22.4) | 0.223 | 43.1 |
| History of malignant neoplasm | 623(22.7) | 303(19.8) | 0.027 | 43.1 |
| CHADS_2_ score | 2.00 [1.00, 3.00] | 2.00 [1.00, 3.00] | <0.001 | 0 |
| CHA_2_DS_2_-VASc score | 3.00 [2.00, 5.00] | 3.00 [2.00, 4.00] | <0.001 | 0.1 |
| Major bleeding | 276(10.1) | 286(6.0) | <0.001 | 0 |
| Clinically relevant non-major bleeding | 1218(44.4) | 948(19.9) | <0.001 | 0 |
| Any bleeding | 1291(47.1) | 1097(23.0) | <0.001 | 0 |
| Ischemic stroke | 87(3.2) | 134(2.8) | 0.406 | 0 |
| Systemic embolism | 68(2.5) | 28(0.6) | <0.001 | 0 |
| Hemorrhagic stroke | 42(1.5) | 67(1.4) | 0.729 | 0 |
| Heart failure admission | 283(10.3) | 123(8.0) | 0.016 | 43.1 |
| All cause death | 187(6.8) | 296(6.2) | 0.316 | 0 |

Data with listwise deletion are expressed as median [interquartile range] or number (percentage). Abbreviations: AF, Atrial fibrillation.

**Table S5. Baseline characteristics of phenotypes identified without prior imputation (sensitivity analysis)**

| **Variable** | **Phenotype 1** | **Phenotype 2** | **Phenotype 3** | **P value** | **Missing (%)** |
| --- | --- | --- | --- | --- | --- |
|  | **N = 1250** | **N = 1127** | **N = 364** |  |  |
| Age | 71.00 [63.00, 77.00] | 75.00 [68.00, 81.00] | 70.50 [59.00, 78.00] | <0.001 | 0 |
| Female sex | 492 (39.4) | 378 (33.5) | 97 (26.6) | <0.001 | 0 |
| AF type |  |  |  | <0.001 | 5.9 |
| Paroxysmal | 828 (69.1) | 559 (53.6) | 215 (63.8) |  |  |
| Persistent | 181 (15.1) | 195 (18.7) | 60 (17.8) |  |  |
| Long standing persistent | 189 (15.8) | 289 (27.7) | 62 (18.4) |  |  |
| Hypertension | 756 (60.5) | 869 (77.1) | 233 (64.0) | <0.001 | 0 |
| Diabetes mellitus | 238 (19.0) | 384 (34.1) | 101 (27.7) | <0.001 | 0 |
| Dyslipidemia | 627 (50.2) | 641 (56.9) | 194 (53.3) | 0.004 | 0 |
| Coronary artery disease | 102 (8.2) | 205 (18.2) | 62 (17.0) | <0.001 | 0 |
| History of stroke | 216 (17.3) | 347 (30.8) | 99 (27.2) | <0.001 | 0 |
| History of heart failure | 165 (13.2) | 541 (48.0) | 261 (71.7) | <0.001 | 0 |
| Liver dysfunction | 237 (19.0) | 327 (29.0) | 97 (26.6) | <0.001 | 0 |
| Hemoglobin, g/dL | 13.6 [12.5, 14.9] | 12.9 [11.3, 14.6] | 13.1[11.5, 14.3] | <0.001 | 2.7 |
| Smoking |  |  |  | <0.001 | 1.9 |
| Never | 662 (54.1) | 499 (45.1) | 132 (36.7) |  |  |
| Past | 426 (34.8) | 489 (44.2) | 156 (43.3) |  |  |
| Current | 136 (11.1) | 118 (10.7) | 72 (20.0) |  |  |
| Creatinine, mg/dL | 0.8 [0.7, 0.9] | 1.0 [0.8, 1.2] | 0.9 [0.7, 1.1] | <0.001 | 2.7 |
| CRP, mg/L | 0.0 [0.0, 0.1] | 0.1 [0.0, 0.3] | 1.3 [0.9, 2.4] | <0.001 | 7 |
| LVDd, mm | 47.0 [44.0, 51.0] | 51.0 [46.0, 57.0] | 48.0 [43.0, 52.0] | <0.001 | 0 |
| IVSTd, mm | 8.0 [8.0, 9.0] | 9.0 [8.0, 11.0] | 9.3 [8.0, 12.0] | <0.001 | 0.3 |
| LVPWTd, mm | 8.0 [8.0, 9.0] | 9.0 [8.0, 11.0] | 9.0 [8.0, 10.0] | <0.001 | 0.4 |
| LVEF, % | 67.00 [61.25, 72.00] | 64.00 [54.00, 70.00] | 46.00 [29.23, 63.00] | <0.001 | 0 |
| LADs, mm | 43.00 [38.00, 47.00] | 47.00 [43.00, 52.00] | 48.00 [42.00, 53.00] | <0.001 | 0 |

Abbreviations: AF, Atrial fibrillation; LVDd, left ventricular diastolic dimension; LVDs, left ventricular systolic dimension; IVSTd, interventricular septum thickness in diastole; LVPWTd, left ventricular posterior wall thickness in diastole; LVEF, left ventricular ejection fraction; LADs, left atrial dimension in systole.

**Table S6. Medications in the DIRECT-Extend registry**

| **Variable** | **Phenotype 1** | **Phenotype 2** | **Phenotype 3** | **P value** | **Missing** |
| --- | --- | --- | --- | --- | --- |
|  | **N = 1565** | **N = 981** | **N = 195** |  |  |
| DOAC type |  |  |  | <0.001 | 0 |
| Dabigatran | 379 (24.2) | 158 (16.1) | 41 (21.0) |  |  |
| Rivaroxaban | 420 (26.8) | 255 (26.0) | 52 (26.7) |  |  |
| Apixaban | 434 (27.7) | 326 (33.2) | 63 (32.3) |  |  |
| Edoxaban | 332 (21.2) | 242 (24.7) | 39 (20.0) |  |  |
| NSAIDs | 63 (4.0) | 49 (5.0) | 8 (4.1) | 0.499 | 0 |
| Antiplatelet therapy | 306 (19.6) | 302 (30.8) | 48 (24.6) | <0.001 | 0 |
| Aspirin | 230 (14.7) | 242 (24.7) | 41 (21.0) | <0.001 | 0 |
| Clopidogrel | 88 (5.6) | 79 (8.1) | 4 (2.1) | 0.002 | 0 |
| Ticlopidine | 4 (0.3) | 4 (0.4) | 1 (0.5) | 0.724 | 0 |
| Cilostazol | 18 (1.2) | 20 (2.0) | 6 (3.1) | 0.053 | 0 |
| Prasugrel | 13 (0.8) | 13 (1.3) | 3 (1.5) | 0.392 | 0 |
| Statin | 486 (31.1) | 372 (37.9) | 53 (27.2) | <0.001 | 0 |
| Proton pump inhibitor | 580 (37.1) | 474 (48.3) | 91 (46.7) | <0.001 | 0 |
| H_2_ blocker | 100 (6.4) | 70 (7.1) | 11 (5.6) | 0.651 | 0 |
| Pgp inhibitor | 195 (12.5) | 149 (15.2) | 28 (14.4) | 0.141 | 0 |

Data with listwise deletion are expressed as number (percentage).

Abbreviations: DOAC, Direct oral anticoagulant; NSAIDs, Non-steroidal anti-inflammatory drugs.

**Table S7 Patients’ characteristics across the 3 phenotypes in the EARNEST-PVI trial**

| **Variable** | **Phenotype 1** | **Phenotype 2** | **Phenotype 3** | **P value** | **Missing (%)** |
| --- | --- | --- | --- | --- | --- |
|  | **N = 328** | **N = 139** | **N = 30** |  |  |
| Age | 66.5 [59.0, 72.0] | 67.0 [60.0, 73.0] | 66.0 [58.5, 70.8] | 0.434 | 0 |
| Female | 91 (27.7) | 25 (18.0) | 5 (16.7) | 0.048 | 0 |
| Body weight, Kg | 66.0 [58.3, 74.0] | 68.4 [61.0, 77.0] | 69.0 [63.1, 74.8] | 0.04 | 0 |
| Body mass index, Kg/m2 | 23.95 [21.85, 26.29] | 24.85 [23.07, 27.42] | 24.42 [22.22, 26.28] | 0.022 | 0.2 |
| Long standing persistent AF | 76 (23.2) | 39 (28.1) | 9 (30.0) | 0.432 | 0 |
| Hypertension | 177 (54.0) | 107 (77.0) | 15 (50.0) | <0.001 | 0 |
| Diabetes mellitus | 39 (11.9) | 39 (28.1) | 7 (23.3) | <0.001 | 0 |
| Dyslipidemia | 139 (42.4) | 71 (51.1) | 17 (56.7) | 0.104 | 0 |
| Vascular disease | 29 (8.8) | 24 (17.3) | 3 (10.0) | 0.03 | 0 |
| History of stroke | 18 (5.5) | 18 (12.9) | 3 (10.0) | 0.021 | 0 |
| History of heart failure | 45 (13.7) | 45 (32.4) | 2 (6.7) | <0.001 | 0 |
| Smoking |  |  |  | 0.585 | 0 |
| Never | 132 (40.2) | 49 (35.3) | 14 (46.7) |  |  |
| Past | 45 (13.7) | 16 (11.5) | 3 (10.0) |  |  |
| Current | 151 (46.0) | 74 (53.2) | 13 (43.3) |  |  |
| Hemoglobin, g/dL | 14.5 [13.6, 15.3] | 14.7 [13.6, 15.6] | 14.6 [13.8, 15.2] | 0.594 | 0.2 |
| Creatinine, mg/dL | 0.8 [0.8, 1.0] | 1.0 [0.9, 1.1] | 0.8 [0.7, 0.9] | <0.001 | 0 |
| CRP, mg/L | 0.1 [0.0, 0.1] | 0.2 [0.1, 0.3] | 1.0 [0.9, 1.8] | <0.001 | 11.3 |
| BNP, pg/mL | 145.0 [91.5, 205.5] | 149.6 [104.6, 247.2] | 133.2 [91.3, 194.4] | 0.151 | 4 |
| LVDd, mm | 46.0 [43.5, 49.0] | 46.5 [43.0, 50.5] | 46.0 [44.0, 49.0] | 0.459 | 0.2 |
| LVDs, mm | 30.0 [27.6, 33.0] | 30.8 [27.0, 36.5] | 30.6 [29.0, 35.0] | 0.219 | 0.2 |
| IVSTd, mm | 9.0 [8.0, 10.0] | 10.0 [9.0, 11.0] | 9.0 [8.9, 11.0] | <0.001 | 0.4 |
| LVPWTd, mm | 9.0 [8.0, 10.0] | 10.0 [9.0, 11.0] | 9.0 [9.0, 11.0] | <0.001 | 0.6 |
| LVEF, % | 72.1 [67.8, 76.0] | 72.4 [60.6, 77.9] | 69.5 [66.3, 72.5] | 0.321 | 0.2 |
| LADs, mm | 42.0 [38.6, 45.0] | 43.0 [40.9, 46.0] | 40.5 [39.0, 42.9] | 0.002 | 0.2 |
| Mitral regurgitation |  |  |  | 0.161 | 0.4 |
| Non or trace | 98 (30.0) | 45 (32.6) | 6 (20.0) |  |  |
| Mild | 157 (48.0) | 70 (50.7) | 22 (73.3) |  |  |
| Moderate-severe | 65 (19.9) | 20 (14.5) | 2 (6.7) |  |  |
| Severe | 7 (2.1) | 3 (2.2) | 0 (0.0) |  |  |
| DR-FLASH score | 3.7±1.2 | 4.1±1.1 | 3.5±1.2 | 0.001 | 0 |
| DR-FLASH score > 3 | 169 (51.5) | 95 (68.3) | 16 (53.3) | 0.003 | 0 |

Data with listwise deletion are expressed as median [interquartile range] or number (percentage).

Abbreviations: LVDd, left ventricular diastolic dimension; LVDs, left ventricular systolic dimension; IVSTd, interventricular septum thickness in diastole; LVPWTd, left ventricular posterior wall thickness in diastole; LVEF, left ventricular ejection fraction; LADs, left atrial dimension in systole.

**Table S8 Extensive Ablation in EARNEST-PVI trial**

|  | **Phenotype 1**  **PVI-Plus**  **(N=160)** | **Phenotype 2**  **PVI-Plus**  **(N=74)** | **Phenotype 3**  **PVI-Plus**  **(N=14)** |
| --- | --- | --- | --- |
| **Linear ablation** | 138 (86.2) | 60 (81.1) | 13 (92.9) |
| Roof line | 137 (99.3) | 60 (100.0) | 113 (100.0) |
| Bottom line | 67 (48.6) | 36 (60.0) | 7 (53.8) |
| Posterior isolation | 67 (48.6) | 36 (60.0) | 7 (53.8) |
| Anterior line | 27 (19.6) | 9 (15.0) | 4 (30.8) |
| Mitral isthmus | 110 (79.7) | 51 (85.0) | 10 (76.9) |
| **CFAE ablation** | 24 (15.0) | 13 (17.6) | 1 (7.1) |

Data with listwise deletion are expressed as number (percentage).

Abbreviations: PVI, pulmonary vein isolation; CFAE, complex fractionated atrial electrogram.

**Figure S1. Bayesian information criterion**

The plots illustrate the association between the Bayesian information criterion (BIC) and number of groups defined by the latent class analysis. In this analysis, we observed a trend where the BIC decreased with an increasing number of clusters. However, with more groups, the clustering becomes less practical for everyday clinical use and interpreting the characteristics of each group becomes challenging. Using the elbow method, we determined that clustering into 3 groups strike a balance, offering an optimal number of clusters for both practical application and interpretability.

**Figure S2. Discriminative power ranking**

Discriminative power of the 16 variables selected by latent class analysis is shown as a bar graph. We computed the discriminative power of each variable which was defined as the logarithm of the ratio between the probability that the variable is relevant for clustering and the variable is irrelevant for clustering.

Abbreviations: CRP, C-reactive protein; IVSTd, interventricular septum thickness in diastole; LVEF, left ventricular ejection fraction; LVDd, left ventricular diastolic dimension; LVPWTd, left ventricular posterior wall thickness in diastole; LADs, left atrial diameter in systole; AF, atrial fibrillation.

**Figure S3. Probabilities of misclassification**

Probability of misclassification in the classified groups by latent class analysis in the derivation cohort is summarized in bar graphs. X axis indicates probabilities of misclassification, while Y axis shows the number of cases.

**Figure S4. Secondary outcomes in the DIRECT-Extend registry**

Kaplan–Meier curves for key secondary endpoints stratified by phenotype

Abbreviations: CRNMB, clinically relevant non-major bleeding
